# Impact of Changing Clinical Practices on Early Blood Gas Analyses in Very Preterm Infants and Their Associated Inpatient Outcomes

**DOI:** 10.3389/fped.2017.00011

**Published:** 2017-02-13

**Authors:** Hongmei Huang, Po-Yin Cheung, Megan O’Reilly, Sylvia van Os, Anne Lee Solevåg, Khalid Aziz, Georg M. Schmölzer

**Affiliations:** ^1^Centre for the Studies of Asphyxia and Resuscitation, Neonatal Research Unit, Royal Alexandra Hospital, Edmonton, AB, Canada; ^2^Department of Pediatrics, Hong Kong University-Shenzhen Hospital, Shenzhen, China; ^3^Department of Pediatrics, University of Alberta, Edmonton, AB, Canada

**Keywords:** infants, delivery room, neonatal resuscitation, blood gas, hypocarbia, hypercarbia, hyperoxia, hypoxia

## Abstract

**Background:**

Early studies suggest an association of abnormal carbon dioxide (PCO_2_) or oxygen (PO_2_) levels with adverse inpatient outcomes in very preterm babies. Recent resuscitation practice changes, such as targeted oxygen therapy, end-expiratory pressure, and rescue surfactant may influence these associations.

**Objective:**

The aim of this study is to assess the range of the initial partial pressures of PCO_2_ and PO_2_ in preterm neonates <33 weeks gestational age after birth and their correlation to inpatient neonatal outcomes.

**Study design:**

This is a prospective observational cohort study of infants <33 weeks gestational age with arterial or venous blood gas analysis performed within the first hour after birth.

**Results:**

One hundred seventy infants (arterial *n* = 75, venous *n* = 95) with mean (SD) gestational age of 28 (3) weeks and birth weight of 1,111 (403) g were included. None of the infants with arterial blood gases had hypocarbia (<30 mmHg), 32 (43%) had normocarbia (30–55 mmHg), and 43 (57%) had hypercarbia (>55 mmHg). Seventeen of the infants with arterial blood gases (22%) had hypoxia (<50 mmHg), 50 (67%) normoxia, and 8 (11%) hyperoxia (>80 mmHg). In infants with venous blood samples, none had venous PCO_2_ < 40 mmHg, 41 (43%) had venous PCO_2_ 40–60 mmHg, and 54 (57%) had venous PCO_2_ > 60 mmHg. Multivariable logistic regression analysis showed no association of low or high PCO_2_ or PO_2_ with death or major inpatient morbidities.

**Conclusion:**

With current resuscitation and stabilization practices, hyperoxia and hypocarbia was uncommon, and hypercarbia occurred frequently. None of these findings correlate with adverse inpatient outcomes or death. Our findings are in direct contrast to published observations using historical practices.

## Introduction

Abnormally high and low levels of carbon dioxide and oxygen in the first few hours or days after birth have been associated with inpatient mortality and morbidities in very preterm babies (1–3). Over a decade ago, Tracy et al. reported hypocarbia and hyperoxia during the resuscitation of preterm infants with severe respiratory distress in the delivery room (DR) (4). At that time, respiratory support in the DR often included (i) self-inflating bags, (ii) no positive end-expiratory pressure (PEEP), (iii) early intubation, and (iv) prophylactic surfactant administration (5, 6). In addition, infants were resuscitated with 100% oxygen, and inspired oxygen concentration was not titrated against oxygen saturation (5, 6). Invasive respiratory support can cause lung injury in preterm infants through several mechanisms including high pressures (barotrauma), hyperinflation and overdistension (volutrauma), alveolar instability due to repeated expansion and collapse (atelectrauma), and the release of inflammatory mediators (biotrauma) (7). Added exposure to high oxygen concentration causes oxidant-mediated lung injury (8). As a result, a variety of lung protective strategies have been introduced in the DR (7, 9).

Practice changes in the DR have included (i) using PEEP during positive-pressure ventilation (PPV) (10–12), (ii) early use of non-invasive respiratory support with continuous positive airway pressure (CPAP) (13), (iii) elective and selective intubation for surfactant administration (14), (iv) initial use of a low inspired oxygen concentration (21–40%) rather than starting with 100% oxygen (15), and (v) targeted oxygen delivery using oxygen saturation reference ranges (16, 17). A recent randomized controlled trial including 88 preterm infants reported less oxygen exposure and oxidative stress with decreased respiratory morbidities when a limited oxygen strategy was used in DR resuscitation, compared to that of a high oxygen strategy (18).

We hypothesize that improved resuscitation practices have led to less variation in blood gas values and thus a reduced contribution of these values to adverse inpatient outcomes. We describe early blood gas measurements (in the first hour after birth) in preterm neonates (<33 weeks gestational age) receiving current DR stabilization techniques to control oxygenation and ventilation. In addition, we evaluate the association of initial partial pressures of carbon dioxide (PCO_2_) and oxygen (PO_2_) and their correlation with inpatient morbidities and mortality.

## Patients and Methods

### Settings

This study was carried out between July 2013 and October 2014 at the Royal Alexandra Hospital, Edmonton, AB, Canada, a tertiary perinatal center admitting approximately 350 infants with a birth weight of <1,500 g annually to the neonatal intensive care unit (NICU). The Neonatal Research Committee, Northern Alberta Neonatal Program, and Health Research Ethics Board, University of Alberta approved the study, and written deferred parental consent was obtained for chart abstraction after delivery. When available, the research team attended deliveries of preterm infants of <33 weeks gestational age in addition to the Resuscitation-Stabilization-Triage team (usually a neonatal nurse, neonatal respiratory therapist, neonatal nurse practitioner or neonatal fellow, and a neonatal consultant). The research team was not involved in the clinical care of the infants.

### Patient Inclusion Criteria

The study was limited to infants <33 weeks gestational age who were part of a randomized controlled trial of lung aeration at birth (http://ClinicalTrials.gov Identifier: NCT01739114). Infants were excluded if there was uncertainty about their gestational age, if they had a congenital abnormality, or if parents refused to give consent.

### DR Stabilization

All practitioners attending deliveries received training on the use of equipment and initiation of resuscitation interventions according to the current Neonatal Resuscitation Program protocol (19). At the Royal Alexandra Hospital, all preterm infants received delayed cord clamping for 60 s if deemed appropriate by the obstetric team (20). If respiratory support was needed, it was initiated with air in babies >28 + 0 weeks gestation and 30% oxygen in babies <28 + 0 weeks and titrated according to the 2010 neonatal resuscitation guidelines (19). Respiratory support was provided using a T-piece device (Giraffe Warmer; GE Health Care, Burnaby, BC, Canada), which is a continuous-flow, pressure-limited device with a built-in manometer and a PEEP valve. The default settings used were a gas flow of 8 L/min, a peak inflation pressure (PIP) of 24 cmH_2_O, and PEEP of 6 cmH_2_O. The clinical team adjusted PIP, PEEP, and fraction of inspired oxygen according to the infant’s need. Respiratory support in the DR was initially provided *via* a round silicone facemask (Fisher & Paykel Healthcare, Auckland, New Zealand). Local protocol dictated predefined intubation criteria if chest compressions were required, heart rate remained <100 beats/min despite 60 s of PPV or prolonged PPV of >10 min. Indications to start CPAP, mask ventilation (PPV), intubation, surfactant administration, chest compressions, and epinephrine administration were decided by Resuscitation-Stabilization-Triage-team according to local protocol.

All included infants had either an umbilical venous and/or arterial catheter placed based on their clinical situation. Local protocol recommends both umbilical arterial and umbilical venous catheters in infants <27 weeks of age and selective use of umbilical venous catheters in infants >27 and <33 weeks of age. Blood gases are routinely drawn within the first hour after birth to assess respiratory status. We also collected outcomes of DR resuscitation and important neonatal morbidities including intraventricular hemorrhage grade 3 and 4 according to Papile et al. (21), periventricular leukomalacia, retinopathy of prematurity, chronic lung disease, and necrotizing enterocolitis.

### Statistical Analysis

Demographics of the infants were recorded. For the analysis of arterial blood gases, we defined PCO_2_ and PO_2_ values as follows: hypocarbia PCO_2_, <30 mmHg; normocarbia PCO_2_, 30–55 mmHg; hypercarbia PCO_2_, >55 mmHg; hypoxia PO_2_, <50 mmHg, normoxia PO_2_, 50–80 mmHg; and hyperoxia PO_2_, >80 mmHg. For venous blood gases, we arbitrarily classified a PCO_2_ < 40 mmHg (hypocarbia), PCO_2_ 40–60 mmHg (normocarbia), and PCO_2_ > 60 mmHg (hypercarbia). For each infant, the first blood gas sample within an hour after birth was analyzed. The data are presented as mean (SD) for normally distributed continuous variables and median (interquartile range) when the distribution was skewed. Data were compared using Student’s *t*-test and Mann–Whitney *U* test for parametric and non-parametric comparisons of continuous variables, respectively, and χ^2^ for categorical variables. *p* Values are two sided, and *p* < 0.05 was considered statistically significant. Binomial logistic regressions (log-linear logistic regression) were performed to ascertain the adjusted and unadjusted effects of PCO_2_ and PO_2_ on intraventricular hemorrhage (grade > 3) and chronic lung disease (defined as oxygen or ventilation requirement at 36 weeks corrected age). Ordinary logistic regression was performed to evaluate the adjusted and unadjusted effects of PCO_2_ and PO_2_ on necrotizing enterocolitis (stage > 2 according to Bell). Statistical analyses were performed with Stata (Intercooled 10, Statacorp, TX, USA) and IBM SPSS Statistics version 24 (IBM Corp.). This was an observational study, and therefore, no sample size calculation before the study was performed. The study was reported according to the Strengthening the Reporting of Observational Studies in Epidemiology (STROBE) statement guidelines (22) – Supplement.

## Results

Demographics of study infants are presented in Table 1. Blood gases were performed in 170 infants (arterial *n* = 75, venous *n* = 95) within first hour (a mean of 47 min) after birth. The infants had mean (SD) gestational age of 28 (3) weeks and birth weight of 1,111 (403) g. Overall, there was a greater number of arterial blood gases collected from preterm infants who were <27 weeks gestation (46 arterial and 7 venous), had lower 1- and 5-min Apgar scores, and were less likely to have delayed cord clamping, compared to infants ≥27 weeks gestation (29 arterial and 88 venous) (*p* < 0.05). There were no differences in gender or Cesarean section rate.

**Table 1:** **Demographics of 170 preterm infants in the study**.

	Arterial blood gas (*n* = 75)	Venous blood gas (*n* = 95)	*p* Values
Gestational age	26 (2)	29 (2)	<0.001
Birth weight	911 (352)	1,262 (374)	<0.001
Male gender^b^	35 (47%)	37 (39%)	0.70
Apgar at 1 min^a^	3 (1–5)	5 (3–7)	<0.001
Apgar at 5 min^a^	6 (5–7)	7 (7–8)	<0.001
Delayed cord clamping^b^	42 (56%)	72 (76%)	0.022
Cesarean section^b^	17 (23%)	24 (25%)	0.746

*Data are presented as mean (SD), median (interquartile range)^a^, and number (%)^b^*.

In the whole cohort, 43% had normocarbia, 57% had hypercarbia, and 3% had hyperoxia.

### Outcomes of Infants with Known Arterial Blood Gas

Of 75 infants who had arterial blood gas drawn within the first hour after birth, none had hypocarbia, 32 (43%) had normocarbia, and 43 (57%) had hypercarbia. A total of 17 (22%) had hypoxia, 50 (67%) had normoxia, and 8 (11%) had hyperoxia. Details regarding DR and secondary outcomes are presented in Tables 2 and 3. Infants with arterial hypercarbia were more likely to be intubated in the 72 h after birth and to receive surfactant; however, no association was seen between hypercarbia, hypocarbia, or normocarbia and secondary outcomes. No associations were found with hypoxia, hyperoxia, or normoxia on initial arterial blood gas and either DR or secondary outcomes.

**Table 2:** **Outcomes of preterm infants with different PaCO_2_ levels**.

	Arterial blood gas (*n* = 75)			Venous blood gas (*n* = 95)		
	Normocarbia (*n* = 32)	Hypercarbia (*n* = 43)	*p* Value	Normocarbia (*n* = 41)	Hypercarbia (*n* = 54)	*p* Value
Mask ventilation in the delivery room	26 (81%)	41 (95%)	0.096	22 (54)	42 (78)	0.008
Intubation in the delivery room	18 (56%)	25 (58%)	0.99	2 (5%)	7 (13%)	
Intubation <72 h	22 (69%)	39 (91%)	0.007	15 (37)	29 (54)	0.08
Surfactant	19 (69%)	39 (91%)	0.001	13 (32)	27 (50)	0.06
Intraventricular hemorrhage grade 3 and 4	5 (16%)	9 (21%)	0.99	0 (0)	2 (4)	0.36
Periventricular leukomalacia	0	2 (5%)	0.46	0 (0)	1 (2)	0.50
Necrotizing enterocolitis	7 (26%)	10 (23%)	0.18	4 (10)	6 (11)	0.51
Retinopathy of prematurity	7 (26%)	12 (34%)	0.64	1 (3)	2 (4)	0.70
Bronchopulmonary dysplasia	14 (51%)	16 (46%)	0.61	6 (16)	5 (10)	0.75
Death before discharge	5 (16%)	8 (19%)	0.71	3 (7)	3 (6)	0.76

*Data are presented as number (%). Bronchopulmonary dysplasia and retinopathy of prematurity are presented as percentage of surviving infants*.

**Table 3:** **Outcomes of preterm infants with different PaO_2_ levels**.

	Arterial blood gas (*n* = 75)			
	Hypoxia (*n* = 17)	Normoxia (*n* = 56)	Hyperoxia (*n* = 2)	*p* Value
Mask ventilation in the delivery room	16 (94%)	49 (88%)	2 (100%)	0.74
Intubation in the delivery room	9 (53%)	33 (59%)	1 (50%)	0.89
Intubation <72 h	14 (82%)	45 (80%)	2 (100%)	0.8
Surfactant	14 (82%)	43 (76%)	2 (100%)	0.71
Intraventricular hemorrhage grade ≥3	5 (29%)	8 (14%)	1 (50%)	0.44
Periventricular leukomalacia	1 (6%)	1 (2%)	0	0.25
Necrotizing enterocolitis	5 (29%)	12 (21%)	0	0.054
Retinopathy of prematurity	2 (14%)	17 (36%)	0	0.45
Bronchopulmonary dysplasia	5 (35%)	24 (51%)	1 (50%)	0.76
Death before discharge	3 (18%)	9 (16%)	1 (50%)	0.47

*Data are presented as number (%). Bronchopulmonary dysplasia and retinopathy of prematurity are presented as percentage of surviving infants*.

### Binomial Logistic Regressions

Binomial logistic regressions were performed to ascertain the adjusted and unadjusted effects of PCO_2_ and PO_2_ on intraventricular hemorrhage, necrotizing enterocolitis, and chronic lung disease. Adjustments were made for sex, gestational age, and birth weight separately and then for all three variables together. Ordinary logistic regression was performed to evaluate the adjusted and unadjusted effects of PCO_2_ and PO_2_ on necrotizing enterocolitis. With respect to the outcome (necrotizing enterocolitis), three categories were considered: “no,” “yes,” and “death from necrotizing enterocolitis.” Sex was excluded from the ordinary logistic regression analysis because of the very small size of male and female infants who died because of necrotizing enterocolitis (3 and 1, respectively). Therefore, the effects of PCO_2_ and PO_2_ on necrotizing enterocolitis were adjusted for gestational age and birth weight separately and then for both together. No statistical significance in *p* values and odds ratios was found for either PCO_2_ or PO_2_ (Table 4).

**Table 4:** **Logistic regression analysis examining adjusted and unadjusted effects of PCO_2_ and PO_2_ on intraventricular hemorrhage, necrotizing enterocolitis, and chronic lung disease**.

Outcome (dependent variable)	Variable	*p* Value	Odds ratio	95% CI for odds ratio
Intraventricular hemorrhage	PCO_2_	0.886	0.998	(0.971–1.026)
	PCO_2_ adjusted for sex	0.825	0.997	(0.969–1.025)
	PCO_2_ adjusted for gestational age	0.928	1.001	(0.972–1.031)
	PCO_2_ adjusted for birth weight	0.900	0.998	(0.971–1.027)
	PCO_2_ adjusted for sex, gestational age, and birth weight	0.898	1.002	(0.972–1.033)
	PO_2_	0.461	0.990	(0.966–1.016)
	PO_2_ adjusted for sex	0.581	0.993	(0.967–1.019)
	PO_2_ adjusted for gestational age	0.913	0.998	(0.971–1.027)
	PO_2_ adjusted for birth weight	0.675	0.994	(0.968–1.022)
	PO_2_ adjusted for sex, gestational age, and birth weight	0.995	1.000	(0.972–1.029)

Necrotizing enterocolitis^a^	PCO_2_	0.814	1.003	(0.978–1.029)
	PCO_2_ adjusted for gestational age	0.951	1.001	(0.974–1.029)
	PCO_2_ adjusted for birth weight	0.691	1.005	(0.979–1.033)
	PCO_2_ adjusted for gestational age, and birth weight	0.780	1.004	(0.977–1.032)
	PO_2_	0.356	1.011	(0.987–1.036)
	PO_2_ adjusted for gestational age	0.707	1.005	(0.979–1.032)
	PO_2_ adjusted for birth weight	0.727	1.005	(0.978–1.032)
	PO_2_ adjusted for gestational age, and birth weight	0.725	1.005	(0.978–1.032)

Chronic lung disease	PCO_2_	0.566	1.008	(0.982–1.035)
	PCO_2_ adjusted for sex	0.551	1.008	(0.982–1.035)
	PCO_2_ adjusted for gestational age	0.891	1.002	(0.973–1.032)
	PCO_2_ adjusted for birth weight	0.608	1.008	(0.979–1.038)
	PCO_2_ adjusted for sex, gestational age, and birth weight	0.932	0.999	(0.968–1.030)
	PO_2_	0.394	1.016	(0.980–1.053)
	PO_2_ adjusted for sex	0.399	1.016	(0.980–1.053)
	PO_2_ adjusted for gestational age	0.509	1.014	(0.973–1.057)
	PO_2_ adjusted for birth weight	0.506	1.014	(0.973–1.057)
	PO_2_ adjusted for sex, gestational age, and birth weight	0.328	1.022	(0.978–1.069)

*^a^Sex was not included in the analysis of necrotizing enterocolitis because of the very small size of male and female infants who died from necrotizing enterocolitis (3 and 1, respectively)*.

## Discussion

Our study observed arterial and venous blood gases in a cohort of very preterm babies in the first hour after birth. By using current resuscitation and stabilization techniques that monitor and control oxygenation and ventilation, we found a low rate of hyperoxia and hyperventilation (hypocarbia), both of which are implicated in tissue injury. We were unable to demonstrate an association of abnormal blood gases with major inpatient morbidity or death.

There are limited data on PCO_2_ and PO_2_ values in preterm infants immediately after birth. A decade ago, Tracy et al. reported that 26% of preterm infants <34 weeks gestation had hypocarbia, 73% had normocarbia, and none had hypercarbia at NICU admission (4). In addition, 38% of infants had hypoxia, and 20% were both hypocarbic and hyperoxic. Kong et al. reported median cord PCO_2_ values of 50 mmHg, with a wide range of PCO_2_ values after NICU admission (23).

The optimal PCO_2_ goal in clinical practice has not been determined; however, both hypocarbia and hypercarbia have been associated with severe intraventricular hemorrhage, periventricular leukomalacia, and bronchopulmonary dysplasia (1–3, 24–26). Both hypocarbia and hypercarbia cause changes to cerebral perfusion–reperfusion, which are associated with brain injury (27). However, these mechanisms may be absent during the first postnatal day, with cerebral blood flow reactivity to PCO_2_ being re-established by the second or third postnatal day in very preterm neonates (27). We examined arterial and venous umbilical blood gases in 170 infants within an hour after birth. We did not observe any hypocarbia and observed normocarbia in 43% and hypercarbia in 57%. In particular, infants with hypercarbia were significantly more likely to be intubated and receive surfactant compared to those with normocarbia (Table 2). We speculate that the significant reduction in hypocarbia was associated with the increased use of CPAP (13) and elective (rather than prophylactic) intubation for surfactant administration (14).

Furthermore, we observed hyperoxia in 3%, hypoxia in 22%, and normoxia in 75% of preterm infants. This is of clinical relevance because oxygen administration at birth leads to an increase in oxidative stress markers (18, 28). The guidelines on the use of oxygen during neonatal resuscitation were revised in 2010, recommending room air (21% oxygen) as the initial gas. This practice was based on animal studies as well as meta-analyses of multiple human studies (16). The situation is a little less clear for preterm infants, even though the potential for harm is greater: cautious use has been recommended in anticipation of future randomized controlled trials. Many centers like ours initiate resuscitation with 21–30% oxygen to avoid hyperoxia (29). Using the published SpO_2_ nomogram of newborn infants in the DR, inspired oxygen concentration is currently titrated to target the oxygen saturation ranges during neonatal resuscitation and stabilization (16). Preterm infants are at an increased risk for oxidative stress and its related injury due to the immaturity of the antioxidative defense mechanisms. Saugstad coined the term “Oxidative Radical Disease of Neonatology” to refer to several free radical-related morbidities in preterm infants including intraventricular hemorrhage, periventricular leukomalacia necrotizing enterocolitis, retinopathy of prematurity, and chronic lung disease (30). Our multiple regressions did not find significant differences in these outcomes in infants with hypoxia versus normoxia. Other clinical practices, such as delayed cord clamping, permissive hypercapnia, and developmentally sensitive care may influence the changes in SpO_2_ and PCO_2_ in the first minutes after birth. These factors should be considered when establishing standard approaches for resuscitation of preterm infants. In addition to the changes in neonatal resuscitation practices, factors such as regionalization of perinatal care and improvements in obstetric care will inevitably influence rates of hypoxia, hyperoxia, hypocarbia, and hypercarbia after birth. It is important that these trends and their potential impact on neonatal outcomes are understood. We believe our study provides interesting and valuable insights into the impact of current practices.

### Limitations

Our greatest limitation was the arbitrary definition of hypoxia, hyperoxia, hypocarbia, and hypercarbia after birth. This was further confounded by the variation in blood gas source (arterial versus venous). Although there is no comprehensive study comparing arterial and venous PCO_2_ in the newborn infants, available data suggest a good correlation between the two measurements (31). In addition, accepting this variability supports the generalizability of our results by mimicking current practice. Our study is also subject to selection bias: data were only collected from infants when the research team was present. As a consequence, the outcomes presented in Tables 2 and 3 and their analyses should be interpreted with caution.

## Conclusion

Measurement and confirmation of hypoxia, hyperoxia, hypocarbia, and hypercarbia after very preterm birth is an inexact science, and prevalence may vary based on clinical practices. Current practices lead to low rates of hyperoxia or hypocarbia. Given the study limitations, no associations were found between early blood gas measurements up to 1 h of age and inpatient morbidity or death.

## Author Contributions

HH was involved in data collection, analysis, and interpretation; wrote the first draft; performed revisions of the drafted article; and approved the final version of the manuscript for submission. P-YC, MO, KA, and GS were involved in the conception and design of the study; involved in data collection, analysis, and interpretation; performed critical revisions of the drafted article; and approved the final version of the manuscript for submission. SO and AS were involved in data collection, analysis, and interpretation; performed critical revisions of the drafted article; and approved the final version of the manuscript for submission.

## Conflict of Interest Statement

The authors declare that the research was conducted in the absence of any commercial or financial relationships that could be construed as a potential conflict of interest.

## Abbreviations

PPV, positive pressure ventilation; DR, delivery room; PEEP, positive end-expiratory pressure; PCO_2_, partial pressure of carbon dioxide in the blood; PO_2_, partial pressure of oxygen in the blood; PIP, peak inflation pressure; IQR, interquartile range.
