# Supplementary material for: Impact of Changing Clinical Practices on Early Blood Gas Analyses in Very Preterm Infants and Their Associated Inpatient Outcomes
Source: Front Pediatr. 2017 Feb 13;5:11. doi: 10.3389/fped.2017.00011 (PMC5303872; doi:10.3389/fped.2017.00011)
Supplement: Supplementary file 1 [file Data_Sheet_1.DOCX]

STROBE Statement—checklist of items that should be included in reports of observational studies

“**Exhaled carbon dioxide and neonatal breathing patterns in preterm infants after birth”**

|  | Item No. | Recommendation | Page  No. | Relevant text from manuscript |
| --- | --- | --- | --- | --- |
| **Title and abstract** | 1 | (*a*) Indicate the study’s design with a commonly used term in the title or the abstract | 1 | Has respiratory support in the delivery room changed over the last decade? |
|  |  | (*b*) Provide in the abstract an informative and balanced summary of what was done and what was found | 6 | **Objective**  To assess initial partial pressures of carbon dioxide (P_CO2_) and oxygen (P_O2_) in preterm neonates <33 weeks gestational age and their correlation to neonatal morbidities and mortality.  **Study design**  A prospective observational study of infants <33 weeks gestational age with arterial or venous blood gas analysis performed within the first hour after birth. Percentage of hypocarbia and hyperoxia was compared to data by *Tracy et al* from 2004.  **Results**  170 infants (arterial n=75, venous n=95) with mean (SD) gestational age 28 (3) weeks and birth weight 1111 (403)g were included. None of the infants with arterial blood gases had hypocarbia, 32 (43%) had normocarbia and 43 (57%) had hypercarbia and 17 (22%) had hypoxia, 56 (75%) normoxia, and 2 (3%) hyperoxia, respectively.  In infants with venous blood samples only none had venous P_CO2_ <40 mmHg, 41 (43%) had venous P_CO2_ 40-60 mmHg and 54 (57%) had venous P_CO2_ >60 mmHg.  **Conclusion**  Compared to data a decade ago no infants had hypocarbia and less infants hyperoxia, how ever more infants had hypercarbia. These changes might be attributed to changes in delivery room management. |
| Introduction | | | |  |
| Background/rationale | 2 | Explain the scientific background and rationale for the investigation being reported | 7 | **Introduction**  A decade ago *Tracy et al* reported that 26% of preterm infants receiving positive pressure ventilation (PPV) in the delivery room (DR) were hypocarbic and 38% had hyperoxia^1^. At that time respiratory support in the DR usually included i) self-inflating bags, ii) no positive end-expiratory pressure (PEEP), iii) early intubation, and iv) prophylactic surfactant administration^2,3^. In addition, infants were resuscitated with 100% oxygen and inspired oxygen concentration was not titrated against oxygen saturation^2,3^. Invasive respiratory support can cause lung injury in preterm infants through several mechanisms including high pressures (barotrauma), hyperinflation and overdistension (volutrauma), alveolar instability due to repeated expansion and collapse (atelectrauma), and through the release of inflammatory mediators (biotrauma)^4^. Added exposure to high oxygen concentration causes oxidant-mediated lung injury^5^. As a result, a variety of lung protective strategies have been introduced in the DR^4,6^.  In the last decade, there have been considerable changes in the respiratory support for preterm infants in the DR with an emphasis on i) using PEEP during PPV^7-9^, ii) early use of non-invasive respiratory support with continuous positive airway pressure (CPAP)^10^, and iii) elective and selective intubation for surfactant administration^11^. Respiratory strategies now include a recommendation for cautious administration of oxygen during resuscitation with the use of low inspired oxygen concentration (21-40%) rather than starting with 100% oxygen^12^. The inspired oxygen concentration is also titrated to target oxygen delivery according to oxygen saturation reference ranges^13,14^. A recent randomized controlled trial including 88 preterm infants reported less oxygen exposure and oxidative stress with decreased respiratory morbidities when a limited oxygen strategy was used in the DR resuscitation, compared to that of a high oxygen strategy^15^. |
| Objectives | 3 | State specific objectives, including any prespecified hypotheses | 8 | We hypothesized that current delivery room stabilization techniques, when compared t those of a decade ago, provide better control of both oxygenation and ventilation as reflected by the frequency of hyperoxia and hypocarbia after resuscitation as well as reducing rates of neonatal morbidities and mortality. The aim of this study was to assess initial partial pressures of carbon dioxide (P_CO2_) and oxygen (P_O2_) in preterm neonates <33 weeks gestational age and their correlation to neonatal morbidities and mortality. |
| Methods | | | |  |
| Study design | 4 | Present key elements of study design early in the paper | 8 |  |
| Setting | 5 | Describe the setting, locations, and relevant dates, including periods of recruitment, exposure, follow-up, and data collection | 8 | **Setting**  This study was carried out between July 2013 and October 2014 at the Royal Alexandra Hospital, Edmonton, Canada, a tertiary perinatal center admitting ~350 infants with a birth weight of <1500g annually to the neonatal intensive care unit. The Neonatal Research Committee, Northern Alberta Neonatal Program, and Health Research Ethics Board, University of Alberta approved the study and written parental consent was obtained for chart abstraction after delivery. When available, the research team attended deliveries of preterm infants <33 weeks gestation in addition to the Resuscitation-Stabilization-Triage team (usually a neonatal nurse, neonatal respiratory therapist, neonatal nurse practitioner or neonatal fellow and a neonatal consultant). The research team was not involved in the clinical care of the infants. |
| Participants | 6 | (*a*) *Cohort study*—Give the eligibility criteria, and the sources and methods of selection of participants. Describe methods of follow-up  *Case-control study*—Give the eligibility criteria, and the sources and methods of case ascertainment and control selection. Give the rationale for the choice of cases and controls  *Cross-sectional study*—Give the eligibility criteria, and the sources and methods of selection of participants | 8-9 | **Patient inclusion criteria**  The study was limited to infants <33 weeks gestational age who were part of a randomized controlled trial of lung aeration at birth. Infants were excluded if there was uncertainty about their gestational age, if they had a congenital abnormality, or if parents refused to give consent. |
|  |  | (*b*) *Cohort study*—For matched studies, give matching criteria and number of exposed and unexposed  *Case-control study*—For matched studies, give matching criteria and the number of controls per case |  |  |
| Variables | 7 | Clearly define all outcomes, exposures, predictors, potential confounders, and effect modifiers. Give diagnostic criteria, if applicable | **9-11** | **Delivery room stabilization**  All practitioners attending deliveries received training on the use of equipment and initiation of resuscitation interventions according to the current Neonatal Resuscitation Program protocol^16^. At the Royal Alexandra Hospital, all preterm infants received delayed cord clamping for 60 seconds if deemed appropriate by the obstetric team^17^. If respiratory support was needed, it was initiated with air in babies >28^+0^ weeks gestation and 30% oxygen in babies <28^+0^ weeks and titrated according to the 2010 neonatal resuscitation guidelines^16^. Respiratory support was provided using a T-piece device (Giraffe Warmer; GE Health Care, Burnaby, Canada), which is a continuous-flow, pressure-limited device with a built-in manometer and a positive end-expiratory pressure valve (PEEP). The default settings used were a gas flow of 8 L/min, a peak inflation pressure (PIP) of 24 cmH_2_O, and PEEP of 6 cmH_2_O. The clinical team adjusted PIP, PEEP, and fraction of inspired oxygen according to the infant’s need. Respiratory support in the delivery room was initially provided via a round silicone facemask (Fisher & Paykel Healthcare, Auckland, New Zealand). Local protocol dictated predefined intubation criteria if chest compressions were required, heart rate remained <100 beats/min despite 60 sec of PPV, or prolonged PPV of >10 min. Indications to start continuous positive airway pressure (CPAP), mask ventilation (PPV), intubation, surfactant administration, chest compressions, and epinephrine administration were decided by Resuscitation-Stabilization-Triage-team according to local protocol.  **Monitoring systems**  IntelliVue MP50 (Philips Healthcare, Philips Electronics Ltd., Markham, ON, Canada) was used to continuously measure heart rate and oxygen saturation. A Masimo Radical pulse oximeter (Masimo Corporation, Irvine CA, USA) probe set at maximum sensitivity and two second averaging was placed around the infant’s right wrist to measure systematic oxygen saturation. Heart rate was measured using three Micro-Premie Leads (Vermed, Bellows Falls, VT, USA). An Invos™ Cerebral/Somatic Oximeter Monitor (Invos 5100, Somanetics Corp., Troy, MI, USA) with the neonatal sensor was used to measure cerebral regional tissue oxygenation expressed as the percentage of oxygenated hemoglobin (oxygenated hemoglobin/total hemoglobin). The transducer was positioned on the left fronto-parietal forehead in each infant regardless of mode of delivery. The sensor on the forehead was secured with a wrap^18^.  **Data collection**  All variables were stored continuously in a multichannel system “ASOR” (ASOR, Rotterdam, Netherlands) for subsequent analysis. Arterial and cerebral tissue oxygen saturation, and heart rate were stored every second, and the sample rate of cerebral regional tissue oxygenation was 8 seconds (0.13Hz).  All included infants had either an umbilical venous and/or arterial catheter placed based on their clinical situation. Local protocol recommends both umbilical arterial and umbilical venous catheter in infants <27 weeks and only umbilical venous catheter >27 weeks. Blood gases are routinely drawn within the first hour after birth to assess respiratory status. We also collected outcomes of delivery room resuscitation and important neonatal morbidities including intraventricular hemorrhage grade III and IV according to Papile^19^, periventricular leucomalacia, retinopathy of prematurity, chronic lung disease, and necrotizing enterocolitis. |
| Data sources/ measurement | 8* | For each variable of interest, give sources of data and details of methods of assessment (measurement). Describe comparability of assessment methods if there is more than one group | 9-11 | **Delivery room stabilization**  All practitioners attending deliveries received training on the use of equipment and initiation of resuscitation interventions according to the current Neonatal Resuscitation Program protocol^16^. At the Royal Alexandra Hospital, all preterm infants received delayed cord clamping for 60 seconds if deemed appropriate by the obstetric team^17^. If respiratory support was needed, it was initiated with air in babies >28^+0^ weeks gestation and 30% oxygen in babies <28^+0^ weeks and titrated according to the 2010 neonatal resuscitation guidelines^16^. Respiratory support was provided using a T-piece device (Giraffe Warmer; GE Health Care, Burnaby, Canada), which is a continuous-flow, pressure-limited device with a built-in manometer and a positive end-expiratory pressure valve (PEEP). The default settings used were a gas flow of 8 L/min, a peak inflation pressure (PIP) of 24 cmH_2_O, and PEEP of 6 cmH_2_O. The clinical team adjusted PIP, PEEP, and fraction of inspired oxygen according to the infant’s need. Respiratory support in the delivery room was initially provided via a round silicone facemask (Fisher & Paykel Healthcare, Auckland, New Zealand). Local protocol dictated predefined intubation criteria if chest compressions were required, heart rate remained <100 beats/min despite 60 sec of PPV, or prolonged PPV of >10 min. Indications to start continuous positive airway pressure (CPAP), mask ventilation (PPV), intubation, surfactant administration, chest compressions, and epinephrine administration were decided by Resuscitation-Stabilization-Triage-team according to local protocol.  **Monitoring systems**  IntelliVue MP50 (Philips Healthcare, Philips Electronics Ltd., Markham, ON, Canada) was used to continuously measure heart rate and oxygen saturation. A Masimo Radical pulse oximeter (Masimo Corporation, Irvine CA, USA) probe set at maximum sensitivity and two second averaging was placed around the infant’s right wrist to measure systematic oxygen saturation. Heart rate was measured using three Micro-Premie Leads (Vermed, Bellows Falls, VT, USA). An Invos™ Cerebral/Somatic Oximeter Monitor (Invos 5100, Somanetics Corp., Troy, MI, USA) with the neonatal sensor was used to measure cerebral regional tissue oxygenation expressed as the percentage of oxygenated hemoglobin (oxygenated hemoglobin/total hemoglobin). The transducer was positioned on the left fronto-parietal forehead in each infant regardless of mode of delivery. The sensor on the forehead was secured with a wrap^18^.  **Data collection**  All variables were stored continuously in a multichannel system “ASOR” (ASOR, Rotterdam, Netherlands) for subsequent analysis. Arterial and cerebral tissue oxygen saturation, and heart rate were stored every second, and the sample rate of cerebral regional tissue oxygenation was 8 seconds (0.13Hz).  All included infants had either an umbilical venous and/or arterial catheter placed based on their clinical situation. Local protocol recommends both umbilical arterial and umbilical venous catheter in infants <27 weeks and only umbilical venous catheter >27 weeks. Blood gases are routinely drawn within the first hour after birth to assess respiratory status. We also collected outcomes of delivery room resuscitation and important neonatal morbidities including intraventricular hemorrhage grade III and IV according to Papile^19^, periventricular leucomalacia, retinopathy of prematurity, chronic lung disease, and necrotizing enterocolitis. |
| Bias | 9 | Describe any efforts to address potential sources of bias | N/A |  |
| Study size | 10 | Explain how the study size was arrived at | N/A |  |

Continued on next page

| Quantitative variables | 11 | Explain how quantitative variables were handled in the analyses. If applicable, describe which groupings were chosen and why | 8 | **Statistical analysis**  Demographics of the infants were recorded. For the analysis of arterial blood gases, we defined P_CO2_ and P_O2_ values according to a previously published study by *Tracy et al*^1^: Hypocarbia P_CO2_ <30mmHg, normocarbia P_CO2_ 30-55mmHg, and hypercarbia P_CO2_ >55mmHg; hypoxia P_O2_ <50mmHg, normoxia P_O2_ 50-100mmHg and hyperoxia P_O2_ >100mmHg. For venous blood gases we classified a P_CO2_ <40mmHg (hypocarbia) P_CO2_ 40-60mmHg (normocarbia) and P_CO2_ >60 mmHg (hypercarbia). The data are presented as mean (standard deviation (SD)) for normally distributed continuous variables and median (interquartile range, (IQR)) when the distribution was skewed. Data were compared using Student’s t-test and Mann-Whitney U test for parametric and nonparametric comparisons of continuous variables, respectively, and χ2 for categorical variables. For the comparison of proportions of the current study and that of *Tracy et al*^1^, z-test was used. P-values are 2-sided and p<0.05 was considered statistically significant. Statistical analyses were performed with Stata (Intercooled 10, Statacorp, Texas, USA). The study was reported according to the STROBE (the Strengthening the Reporting of Observational Studies in Epidemiology statement) guidelines^20^. |
| --- | --- | --- | --- | --- |
| Statistical methods | 12 | (*a*) Describe all statistical methods, including those used to control for confounding | 8 | **Statistical analysis**  Demographics of the infants were recorded. For the analysis of arterial blood gases, we defined P_CO2_ and P_O2_ values according to a previously published study by *Tracy et al*^1^: Hypocarbia P_CO2_ <30mmHg, normocarbia P_CO2_ 30-55mmHg, and hypercarbia P_CO2_ >55mmHg; hypoxia P_O2_ <50mmHg, normoxia P_O2_ 50-100mmHg and hyperoxia P_O2_ >100mmHg. For venous blood gases we classified a P_CO2_ <40mmHg (hypocarbia) P_CO2_ 40-60mmHg (normocarbia) and P_CO2_ >60 mmHg (hypercarbia). The data are presented as mean (standard deviation (SD)) for normally distributed continuous variables and median (interquartile range, (IQR)) when the distribution was skewed. Data were compared using Student’s t-test and Mann-Whitney U test for parametric and nonparametric comparisons of continuous variables, respectively, and χ2 for categorical variables. For the comparison of proportions of the current study and that of *Tracy et al*^1^, z-test was used. P-values are 2-sided and p<0.05 was considered statistically significant. Statistical analyses were performed with Stata (Intercooled 10, Statacorp, Texas, USA). The study was reported according to the STROBE (the Strengthening the Reporting of Observational Studies in Epidemiology statement) guidelines^20^. |
|  |  | (*b*) Describe any methods used to examine subgroups and interactions | N/A |  |
|  |  | (*c*) Explain how missing data were addressed | N/A |  |
|  |  | (*d*) *Cohort study*—If applicable, explain how loss to follow-up was addressed  *Case-control study*—If applicable, explain how matching of cases and controls was addressed  *Cross-sectional study*—If applicable, describe analytical methods taking account of sampling strategy |  |  |
|  |  | (*e*) Describe any sensitivity analyses | N/A |  |
| Results | | | | |
| Participants | 13* | (a) Report numbers of individuals at each stage of study—eg numbers potentially eligible, examined for eligibility, confirmed eligible, included in the study, completing follow-up, and analysed | 12 | Demographics of study infants are presented in Table 1. A total of 170 infants (arterial n=75, venous n=95) with mean (SD) gestational age 28 (3) weeks and birth weight 1111 (403)g were included in the study. Overall more arterial blood gas were collected from preterm infants with <27 weeks gestation (46 arterial and 7 venous), compared to infants ≥27 weeks gestation (29 arterial and 88 venous)(p<0.05). The infants with arterial blood gas collected were more immature in gestation, were lower in birth weight, had lower Apgar scores at 1^st^ and 5^th^ minutes of life, and had less delayed cord clamping, compared to those of infants with venous blood gas collected, with no differences in gender and cesarean section rate (Table 1). Compared to *Tracy et al* a normocarbia significantly decreased to 43% (p<0.005), while hypercarbia significantly increased to 57% (p<0.001). A significant reduction in hyperoxia to 3% (p<0.001) was also observed. Physiological observations of oxygen saturation, heart rate, and cerebral tissue oxygenation along with fraction of inspired oxygen over the first 60minutes after birth in infants with normocarbia, normoxia, hypercarbia, and hyperoxia are presented in Figure 1. |
|  |  | (b) Give reasons for non-participation at each stage | N/A |  |
|  |  | (c) Consider use of a flow diagram | N/A |  |
| Descriptive data | 14* | (a) Give characteristics of study participants (eg demographic, clinical, social) and information on exposures and potential confounders | Table 1 |  |
|  |  | (b) Indicate number of participants with missing data for each variable of interest | N/A |  |
|  |  | (c) *Cohort study*—Summarise follow-up time (eg, average and total amount) | N/A |  |
| Outcome data | 15* | *Cohort study*—Report numbers of outcome events or summary measures over time |  |  |
|  |  | *Case-control study—*Report numbers in each exposure category, or summary measures of exposure | *12-14* | **Outcomes of infants with arterial blood gas collected**  A total of 75 infants had arterial blood gas drawn within the first hour after birth. None of them had hypocarbia, 32 (43%) had normocarbia and 43 (57%) had hypercarbia. A total of 17 (22%) had hypoxia, 56 (75%) normoxia, and 2 (3%) hyperoxia, respectively.  *Arterial Partial Pressure of Carbon Dioxide*  Of the 75 infants, 30/32 and 40/43 infants who had normocarbia and hypercarbia, respectively, received PPV in the DR (p=0.47). Compared to those with normocarbia, significantly more infants with hypercarbia required intubation and surfactant administration (22/32 vs. 40/43 and 19/32 vs. 40/43; both p<0.005, respectively). Five of 32 and 8/43 infants with normocarbia and hypercarbia respectively died during admission (p=0.71). There were no significant difference in the proportions of infants with normocarbia and hypercarbia regarding the development of intraventricular hemorrhage grade III and IV, periventricular leucomalacia, necrotizing enterocolitis, retinopathy of prematurity and bronchopulmonary dysplasia (Table 2).  *Arterial Partial Pressure of Oxygen*  Of the 75 infants, 16/17, 49/56 and 2/2 infants who had hypoxia, normoxia and hyperoxia, respectively, received PPV in the DR with no differences between groups (p=0.74). Similar amount of infants were intubation and received surfactant administration in all groups (Table 3). There were no significant difference in the proportions of infants with hypoxia or normoxia regarding the mortality, development of intraventricular hemorrhage grade III and IV, periventricular leucomalacia, necrotizing enterocolitis, retinopathy of prematurity and bronchopulmonary dysplasia (Table 3). In the hyperoxia group we only had two infants and therefore no analysis could be done (Table 3).  *Outcomes of infants with venous blood gas collected*  A total of 95 infants had venous blood gas drawn within the first hour after birth with a median (IQR) P_CO2_ of 56 (50-65) mm Hg. None of them had venous P_CO2_ <40 mmHg, 41 (43%) had venous P_CO2_ 40-60 mmHg and 54 (57%) had venous P_CO2_ >60 mmHg. Significantly less infants whom had venous P_CO2_ 40-60 mmHg received PPV in the DR compared to infants with venous P_CO2_ >60 mmHg (22/41 vs. 42/54, p<0.01). There were modest trends for fewer infants with venous P_CO2_ 40-60 mmHg requiring intubation and surfactant administration, compared to infants with venous P_CO2_ >60 mmHg (Table 2). There were no significant differences between two groups of infants regarding mortality, the development of intraventricular hemorrhage grade III and IV, periventricular leucomalacia necrotizing enterocolitis, retinopathy of prematurity and bronchopulmonary dysplasia (Table 2). |
|  |  | *Cross-sectional study—*Report numbers of outcome events or summary measures |  |  |
| Main results | 16 | (*a*) Give unadjusted estimates and, if applicable, confounder-adjusted estimates and their precision (eg, 95% confidence interval). Make clear which confounders were adjusted for and why they were included |  |  |
|  |  | (*b*) Report category boundaries when continuous variables were categorized | 12-14 | **Outcomes of infants with arterial blood gas collected**  A total of 75 infants had arterial blood gas drawn within the first hour after birth. None of them had hypocarbia, 32 (43%) had normocarbia and 43 (57%) had hypercarbia. A total of 17 (22%) had hypoxia, 56 (75%) normoxia, and 2 (3%) hyperoxia, respectively.  *Arterial Partial Pressure of Carbon Dioxide*  Of the 75 infants, 30/32 and 40/43 infants who had normocarbia and hypercarbia, respectively, received PPV in the DR (p=0.47). Compared to those with normocarbia, significantly more infants with hypercarbia required intubation and surfactant administration (22/32 vs. 40/43 and 19/32 vs. 40/43; both p<0.005, respectively). Five of 32 and 8/43 infants with normocarbia and hypercarbia respectively died during admission (p=0.71). There were no significant difference in the proportions of infants with normocarbia and hypercarbia regarding the development of intraventricular hemorrhage grade III and IV, periventricular leucomalacia, necrotizing enterocolitis, retinopathy of prematurity and bronchopulmonary dysplasia (Table 2).  *Arterial Partial Pressure of Oxygen*  Of the 75 infants, 16/17, 49/56 and 2/2 infants who had hypoxia, normoxia and hyperoxia, respectively, received PPV in the DR with no differences between groups (p=0.74). Similar amount of infants were intubation and received surfactant administration in all groups (Table 3). There were no significant difference in the proportions of infants with hypoxia or normoxia regarding the mortality, development of intraventricular hemorrhage grade III and IV, periventricular leucomalacia, necrotizing enterocolitis, retinopathy of prematurity and bronchopulmonary dysplasia (Table 3). In the hyperoxia group we only had two infants and therefore no analysis could be done (Table 3).  *Outcomes of infants with venous blood gas collected*  A total of 95 infants had venous blood gas drawn within the first hour after birth with a median (IQR) P_CO2_ of 56 (50-65) mm Hg. None of them had venous P_CO2_ <40 mmHg, 41 (43%) had venous P_CO2_ 40-60 mmHg and 54 (57%) had venous P_CO2_ >60 mmHg. Significantly less infants whom had venous P_CO2_ 40-60 mmHg received PPV in the DR compared to infants with venous P_CO2_ >60 mmHg (22/41 vs. 42/54, p<0.01). There were modest trends for fewer infants with venous P_CO2_ 40-60 mmHg requiring intubation and surfactant administration, compared to infants with venous P_CO2_ >60 mmHg (Table 2). There were no significant differences between two groups of infants regarding mortality, the development of intraventricular hemorrhage grade III and IV, periventricular leucomalacia necrotizing enterocolitis, retinopathy of prematurity and bronchopulmonary dysplasia (Table 2). |
|  |  | (*c*) If relevant, consider translating estimates of relative risk into absolute risk for a meaningful time period |  |  |

Continued on next page

| Other analyses | 17 | Report other analyses done—eg analyses of subgroups and interactions, and sensitivity analyses | 12-14 | **Outcomes of infants with arterial blood gas collected**  A total of 75 infants had arterial blood gas drawn within the first hour after birth. None of them had hypocarbia, 32 (43%) had normocarbia and 43 (57%) had hypercarbia. A total of 17 (22%) had hypoxia, 56 (75%) normoxia, and 2 (3%) hyperoxia, respectively.  *Arterial Partial Pressure of Carbon Dioxide*  Of the 75 infants, 30/32 and 40/43 infants who had normocarbia and hypercarbia, respectively, received PPV in the DR (p=0.47). Compared to those with normocarbia, significantly more infants with hypercarbia required intubation and surfactant administration (22/32 vs. 40/43 and 19/32 vs. 40/43; both p<0.005, respectively). Five of 32 and 8/43 infants with normocarbia and hypercarbia respectively died during admission (p=0.71). There were no significant difference in the proportions of infants with normocarbia and hypercarbia regarding the development of intraventricular hemorrhage grade III and IV, periventricular leucomalacia, necrotizing enterocolitis, retinopathy of prematurity and bronchopulmonary dysplasia (Table 2).  *Arterial Partial Pressure of Oxygen*  Of the 75 infants, 16/17, 49/56 and 2/2 infants who had hypoxia, normoxia and hyperoxia, respectively, received PPV in the DR with no differences between groups (p=0.74). Similar amount of infants were intubation and received surfactant administration in all groups (Table 3). There were no significant difference in the proportions of infants with hypoxia or normoxia regarding the mortality, development of intraventricular hemorrhage grade III and IV, periventricular leucomalacia, necrotizing enterocolitis, retinopathy of prematurity and bronchopulmonary dysplasia (Table 3). In the hyperoxia group we only had two infants and therefore no analysis could be done (Table 3).  *Outcomes of infants with venous blood gas collected*  A total of 95 infants had venous blood gas drawn within the first hour after birth with a median (IQR) P_CO2_ of 56 (50-65) mm Hg. None of them had venous P_CO2_ <40 mmHg, 41 (43%) had venous P_CO2_ 40-60 mmHg and 54 (57%) had venous P_CO2_ >60 mmHg. Significantly less infants whom had venous P_CO2_ 40-60 mmHg received PPV in the DR compared to infants with venous P_CO2_ >60 mmHg (22/41 vs. 42/54, p<0.01). There were modest trends for fewer infants with venous P_CO2_ 40-60 mmHg requiring intubation and surfactant administration, compared to infants with venous P_CO2_ >60 mmHg (Table 2). There were no significant differences between two groups of infants regarding mortality, the development of intraventricular hemorrhage grade III and IV, periventricular leucomalacia necrotizing enterocolitis, retinopathy of prematurity and bronchopulmonary dysplasia (Table 2). |
| --- | --- | --- | --- | --- |
| Discussion | | | | |
| Key results | 18 | Summarise key results with reference to study objectives | 14-15 | There is limited data on P_CO2_ and P_O2_ values in preterm infants immediately after birth. Two small observational data reported P_CO2_ values within the first hour after birth^1,21^. A decade ago *Tracy at el* reported that 26% of preterm infants <34 weeks gestation had hypocarbia, while 73% had normocarbia and none had hypercarbia at NICU admission. In addition, 38% of infants had hypoxia, and 20% were both hypocarbic and hyperoxic^1^. *Kong et al* reported median cord P_CO2_ values of 50mmHg, with a wide range P_CO2_ values after NICU admission^21^. The optimal P_CO2_ goal in clinical practice has not been determined, however both hypocarbia and hypercarbia have been both associated with severe intraventricular hemorrhage, periventricular leukomalacia, and bronchopulmonary dysplasia^22-27^. Both hypocarbia and hypercarbia causes changes to cerebral perfusion–reperfusion, which are associated with brain injury^28^. However, these mechanisms are absent during the first postnatal day, with cerebral blood flow reactivity to P_CO2_ being re-established by the second or third postnatal day in very preterm neonates^28^. Using the same criteria as *Tracy et al* we examined arterial and venous umbilical blood gases in 170 infants within an hour after birth. We did not observe any hypocarbia but a significant decrease in normocarbia to 43% (p<0.005) and increase in hypercarbia to 57% (p<0.001). In particular, the infants with hypercarbia in arterial samples were significantly more often intubated and received surfactant compared to normocarbia (Table 2). The significant reduction in hypocarbia is associated with change in respiratory support with increased used of CPAP^10^ and elective intubation with surfactant administration^11^.  Using the same criteria as *Tracy et al* we observed a significant reduction in hyperoxia to 3% (p<0.001), with 22% and 75% of preterm infants with hypoxia and normoxia, respectively. |
| Limitations | 19 | Discuss limitations of the study, taking into account sources of potential bias or imprecision. Discuss both direction and magnitude of any potential bias | 16 | **Limitations**  In the current study the blood gas source varied among infants to improve generalizability of our results by mimicking current practice. Although, there is no study comparing arterial and venous P_CO2_ in the newborn infants, available data suggests a good correlation between the two measurements^31^. In addition, we only present data of infants where the research team was present and therefore the secondary outcomes presented in Table 2 and Table 3 should be interpreted with caution. |
| Interpretation | 20 | Give a cautious overall interpretation of results considering objectives, limitations, multiplicity of analyses, results from similar studies, and other relevant evidence | 16 | Changes in resuscitation strategy over the last decade changed initial blood gas parameters causing more hypercarbia and less hypocarbia and hyperoxia. |
| Generalisability | 21 | Discuss the generalisability (external validity) of the study results | 16 | Changes in resuscitation strategy over the last decade changed initial blood gas parameters causing more hypercarbia and less hypocarbia and hyperoxia. |
| Other information | |  | | |
| Funding | 22 | Give the source of funding and the role of the funders for the present study and, if applicable, for the original study on which the present article is based | 2 | MOR is supported by a Molly Towell Perinatal Research Foundation Fellowship. ALS I supported by the Canadian Institute of Health Research (MOP299116) and the South-Eastern Norway Regional Health Authority. GMS is a recipient of the Heart and Stroke Foundation/University of Alberta Professorship of Neonatal Resuscitation and Heart and Stroke Foundation Canada Research Scholar. |

*Give information separately for cases and controls in case-control studies and, if applicable, for exposed and unexposed groups in cohort and cross-sectional studies.

**Note:** An Explanation and Elaboration article discusses each checklist item and gives methodological background and published examples of transparent reporting. The STROBE checklist is best used in conjunction with this article (freely available on the Web sites of PLoS Medicine at http://www.plosmedicine.org/, Annals of Internal Medicine at http://www.annals.org/, and Epidemiology at http://www.epidem.com/). Information on the STROBE Initiative is available at www.strobe-statement.org.
